# Status of water, sanitation, and hygiene and standard precautions in healthcare facilities and its relevance to COVID-19 in Afghanistan

**DOI:** 10.1265/ehpm.21-00272

**Published:** 2022-02-23

**Authors:** Sharifullah Alemi, Keiko Nakamura, Kaoruko Seino, Shafiqullah Hemat

**Affiliations:** 1 Department of Global Health Entrepreneurship, Division of Public Health, Tokyo Medical and Dental University, Tokyo, Japan; 2 Ministry of Public Health, Kabul, Afghanistan

**Keywords:** COVID-19, Standard precautions, Water, Hygiene, Sanitation

## Abstract

**Background:**

To protect the health and safety of healthcare workers (HCWs), it is essential to ensure the provision of sustainable water, sanitation, and hygiene (WASH) services and standard precautions in healthcare facilities (HCF). The objectives of this short communication were 1) to assess the availability of WASH services and standard precautions in HCFs in seven provinces in Afghanistan before the COVID-19 pandemic, and 2) to elucidate the relevance of these patterns with the number of reported HCW infections from COVID-19 in the mentioned provinces.

**Methods:**

We analyzed secondary data from the 2018-19 Afghanistan Service Provision Assessment survey, which included 142 public and private HCFs in seven major provinces in Afghanistan. Data on COVID-19 cases were obtained from the Afghanistan Ministry of Public Health Data Warehouse. Weighted prevalence of WASH services and standard precautions were calculated using frequencies and percentages. ArcGIS maps were used to visualize the distribution of COVID-19 cases, and scatter plots were created to visualize the relevance of WASH services and standard precautions to COVID-19 cases in provinces.

**Results:**

Of the 142 facilities surveyed, about 97% had improved water sources, and over 94% had improved toilet for clients. Overall, HCFs had limited availability of hygiene services and standard precautions, which was lower in private than public facilities. More than half of the facilities had safe final disposal and appropriate storage of sharps and medical waste. Of the seven provinces, Herat province had the highest cumulative COVID-19 case rate among HCWs per 100,000 population and reported lower availability of WASH services and standard precautions in HCFs compared to other provinces.

**Conclusion:**

Our findings show disparities in the availability of WASH services and standard precautions in public and private facilities. Private facilities had a lower availability of hygiene services and standard precautions than public facilities. Provinces with higher availability of WASH services and standard precautions in HCFs had a lower cumulative COVID-19 case rate among HCWs per 100,000 population. Pre-pandemic preparation of adequate WASH services and standard precautions in HCFs could be potentially important in combating infectious disease emergence.

**Supplementary information:**

The online version contains supplementary material available at https://doi.org/10.1265/ehpm.21-00272.

## Introduction

Provision of safe and sustainable water, sanitation, and hygiene (WASH) services and practicing standard precautions is essential to prevent infection transmission among healthcare workers (HCWs), staff, patients, and clients of healthcare facilities (HCFs) [1, 2]. Ensuring recommended hygienic and environmental measures in HCFs helps protect the health and prevent person-to-person transmission of healthcare-associated infections during outbreaks of infectious diseases, including severe acute respiratory syndrome coronavirus-2 (SARS-CoV-2) induced Coronavirus disease (COVID-19) [1]. Exposure to microorganisms in healthcare settings occurs due to poor sanitary and hygienic conditions and inadequate standard precautions [2], which are preventable. Proper WASH services and waste management act as a barricade to COVID-19 transmission in HCFs [3]. The prevalence of healthcare-acquired infections is estimated to be 15.5 per 100 patients in developing countries [4]. Patients and visitors can develop infections during their stay or visit to an HCF, and the number of infections increases dramatically during a public health crisis. A large proportion of infectious disease cases are attributed to unsafe healthcare settings [5]. The global community will not reach the 2030 Sustainable Development Goal (SDG) targets for universal access to WASH (SDG 6.1 and 6.2) and universal health coverage (SDG 3.8) goals without meeting the indicators of WASH services in HCFs by 2030 [6]. A comprehensive assessment of environmental conditions (including WASH services and waste management) and availability of standard precautions in HCFs of 78 low- and middle-income countries (LMICs) revealed that most of the HCFs assessed had insufficient environmental conditions and basic standard precautions for preventing transmission of infections [2]. The evaluation focused on collecting further data in LMICs to explore the scope and distribution of inadequate coverage [2]. Furthermore, it is necessary to undertake an HCF readiness assessment to evaluate the preparedness of HCFs and improve response capacity. Data from an HCF assessment in 54 LMICs identified large disparities in the provision of WASH services and the availability of poor environmental conditions in HCFs [7]. Therefore, HCFs require a comprehensive preparedness and response plan to meet public health objectives for the prevention and control of infections [8].

Standard precautions include measures to break the chain of infections that result from exposure to contaminated body fluids in healthcare settings. These measures are considered essential for the protection of HCWs from infections due to contamination in HCFs [9]. Standard precautions are also necessary for all patients and HCF clients, whether they show symptoms or not. These practices include engaging in frequent hand hygiene with soap and water or alcohol-based hand rub, wearing medical masks, using personal protective equipment (PPE), safely managing sharps waste and medical waste, disinfecting the surfaces and equipment, and regularly cleaning the environment in the healthcare setting [1]. Frequent hand hygiene and using face masks are key control measures that are widely recognized to reduce the risk of COVID-19 infection by interrupting virus transmission [10–12]. The low availability of the standard precautions increases the vulnerability of healthcare users and staff to infections. Consequently, the risk of infection transmission increases during infectious disease outbreaks. In parallel with this, efforts to combat such outbreaks increase the demand for WASH service availability and the use of standard precautions.

Understanding the potential drivers of infectious disease emergence before an emergency incident could be potentially critical in developing effective preparedness and response plan and preventing pandemics [12]. The pre-pandemic assessment of WASH services and standard precautions contribute to identifying the gaps in infection prevention and control (IPC) measures and establishing a firm response mechanism to tackle the pandemic effectively. An integrative review on the role of WASH in reducing COVID-19 transmission suggested that adequate WASH services should be integrated into response and recovery plans and with other relevant sectors [12]. Long-struggling Afghanistan also experienced the surge of COVID-19 and faced a crisis in early 2020 [13]. Several public health measures were put in place to curb the spread of the virus. However, hundreds of front-line HCWs across Afghanistan remain at a high risk of becoming infected with the virus, and dozens of them lost their lives fighting the pandemic. Little is known about the status of environmental conditions and availability of standard precautions in HCFs before the COVID-19 pandemic in Afghanistan. Therefore, this study aimed to 1) assess the availability of WASH services and standard precautions in HCFs in seven provinces in Afghanistan before the COVID-19 pandemic, and 2) elucidate the relevance of these patterns with the number of reported HCW infections from COVID-19 in the mentioned provinces. The findings from this study are expected to provide insights that have relevance for policy and practice.

## Methods

### Data

**Fig. 1:**
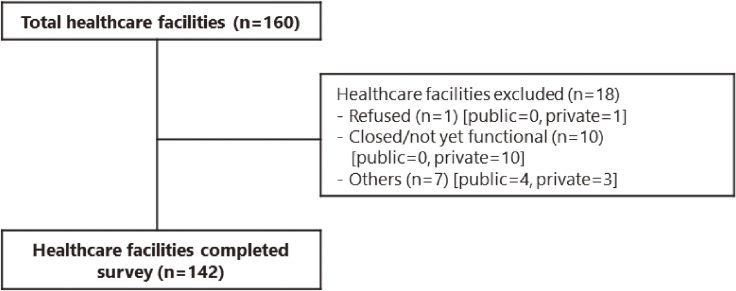
Flowchart of healthcare facilities selected for the analysis

The Afghanistan Service Provision Assessment (AfSPA) survey is a cross-sectional facility survey conducted in 2018–19. The survey collected information on the availability of essential healthcare services and facilities’ general readiness to provide quality health services. The survey included public and private HCFs in urban areas of seven major and most developed provinces in Afghanistan: Balkh, Herat, Kabul, Kandahar, Kunduz, Nangarhar, and Paktya. In six provinces excluding Kabul, no sampling was involved, and all 12 public and 37 private hospitals plus 52 private clinics were included in the sample. In Kabul province, all 26 public and 20 private hospitals were included; however, of the 84 private clinics, 13 clinics were randomly selected for inclusion in the survey to normalize the total number of private clinics at the national level and provide nationally representative results according to HCF type. Of the 160 HCFs visited during the assessment, 18 facilities were permanently closed, unreachable, or refused to participate. Data were successfully collected from a total of 142 facilities (Fig. 1). AfSPA survey used five main types of assessment tools that include facility inventory, health provider interviews, client observation protocols, exit interviews, and country-specific questionnaires. These validated assessment tools are widely used to assess the availability and readiness of HCFs in many countries. For this report, the data from the facility inventory dataset were analyzed. Variables for availability and readiness were identified according to the service availability and readiness assessment (SARA) manual [14]. The AfSPA datasets are available on the Demographic and Health Survey (DHS) program website (https://dhsprogram.com/data/available-datasets.cfm) [15]. Data on COVID-19 cases among HCWs were also analyzed. Data were obtained from the Ministry of Public Health (MoPH) Data Warehouse, which includes the period from the start of the pandemic (February 24, 2020) to November 03, 2020.

### Statistical analysis

Data were analyzed using Stata software version 15.1 and R software version 4.1.2. Weighted prevalence of WASH services and standard precautions were calculated using frequencies and percentages. A sampling weight was applied for the distribution of the HCFs by province to represent their contribution to the total number of HCFs in the survey target areas and ensure the actual representation of the results across the country. Scatter plots were created using the ‘ggplot’ function in R software to visualize the relationship between total COVID-19 infected HCWs per 100,000 population and WASH indicators and standard precautions, as well as to visualize the distribution of total COVID-19 cases (excluding infected HCWs) and total infected HCWs per 100,000 population per province. Geospatial distribution maps were generated using ArcGIS software version 10.7 (ESRI, Redlands, CA, USA). The spatial distribution of total reported COVID-19 cases and infected HCWs according to the total population and province was analyzed and presented in maps.

## Results

### General overview of the surveyed facilities

Table 1 summarizes the characteristics of HCFs. Of the 142 facilities surveyed, 77 (54%) were in Kabul province. Most of the facilities (53.7%) were private clinics, and the private sector managed 84% of the facilities.

**Table 1:** Distribution of surveyed healthcare facilities by background characteristics, N = 142

	***n* (%) of facilities surveyed**	

**Variable**	**Weighted**	**Unweighted**
**Facility location**		
Balkh	10 (7.0)	15 (10.6)
Herat	19 (13.1)	27 (19.0)
Kabul	77 (54.0)	52 (36.6)
Kandahar	11 (7.5)	11 (7.8)
Kunduz	5 (3.8)	8 (5.6)
Nangarhar	15 (10.8)	21 (14.8)
Paktya	5 (3.8)	8 (5.6)
**Facility type**		
Regional/national hospital	6 (4.2)	9 (6.3)
Provincial hospital	1 (0.9)	2 (1.4)
Special hospital	17 (11.8)	25 (17.6)
Private hospital	42 (29.4)	58 (40.9)
Private clinic	76 (53.7)	48 (33.8)
**Managing authority**		
Public/government	23 (16.0)	34 (23.9)
Private (private not-for-profit/private for-profit)	119 (84.0)	108 (76.1)

**Note.** Data were obtained from the AfSPA survey 2018–19.

### Availability of WASH services and standard precautions in general outpatient areas and healthcare waste management

Table 2 presents the status of WASH services and standard precautions observed in general outpatient areas and healthcare waste management in public and private facilities. Of the 142 facilities, around 97% of public and private facilities reported having an improved water source, and 94.1% of public and 97.7% of private facilities reported having improved toilets for clients. Water was available in the clients’ toilets of 85.3% and 88% of public and private facilities, respectively. Only 35.3% of public and 39.6% of private facilities had handwashing soap, and 17.6% of public and 18.9% of private facilities had cleansing materials other than soap in the clients’ toilets. About three-quarters (73%) of public and private facilities had a separate toilet for female clients. Most of the public (64.7%) and private facilities (59.8%) had water, and 29.4% of public and 25% of private facilities had handwashing soap in the female toilets. Cleansing materials were available in the female toilets of 20.6% public and 12.3% private facilities.

**Table 2:** Availability of water, sanitation and hygiene services and standard precautions in general outpatient areas, and healthcare waste management by managing authority, N = 142

	***Weighted n* (%) of facilities surveyed**	

**Variable**	**Public (*n* = 23)**	**Private (*n* = 119)**
**Improved water source^a^**	22 (97.1)	116 (97.3)
**Client toilets**		
Improved toilets^b^	21 (94.1)	117 (97.7)
Water available	19 (85.3)	105 (88.0)
Handwashing soap available	8 (35.3)	47 (39.6)
Cleansing materials available	4 (17.6)	23 (18.9)
**Female toilets**		
Separate female toilets	16 (72.7)	87 (73.0)
Water available	15 (64.7)	71 (59.8)
Handwashing soap available	7 (29.4)	30 (25.0)
Cleansing materials available	5 (20.6)	15 (12.3)
**Hygienic conditions, observed**		
Running water	19 (82.3)	79 (66.4)
Handwashing soap	14 (61.8)	67 (56.4)
Alcohol-based hand disinfectant	12 (52.9)	57 (48.2)
Disinfectant (environmental, e.g., chlorine)	15 (67.6)	76 (63.3)
Guidelines for standard precautions	4 (17.6)	18 (14.8)
**Personal protective equipment, observed**		
Sterile latex gloves	16 (70.6)	79 (66.5)
Medical/surgical masks	15 (64.7)	59 (49.2)
Gowns/aprons	14 (61.8)	52 (44.0)
Eye protection (goggles, face shields)	3 (11.8)	6 (5.5)
**Healthcare waste management, observed**		
Safe final disposal of sharps waste	13 (58.8)	74 (61.5)
Safe final disposal of medical waste	17 (73.5)	79 (66.7)
Appropriate storage of sharps waste	14 (61.8)	62 (52.2)
Appropriate storage of medical waste	15 (67.6)	80 (66.7)

**Note.** Data were obtained from the AfSPA survey 2018-19. ^a^Water from improved sources (protected from outside contamination): on-site piped water, public taps/standpipes, tube wells/boreholes, protected dug wells, protected springs and rain water. ^b^Toilets that hygienically separate human excreta from human, animal and insect contact and include flush/pour-flush toilets connected to a sewer, septic tank, or pit; ventilated improved pit latrine; pit latrine with slab and composting toilet.

Public facilities had higher availability of running water, handwashing soap, alcohol-based hand disinfectant, environmental disinfectant, and guidelines for standard precautions than private facilities (82.3% vs. 66.4%; 61.8% vs. 56.4%; 52.9% vs. 48.2%; 67.6% vs. 63.3%; and 17.6% vs. 14.8%, respectively). Similarly, a higher proportion of public facilities had personal protective equipment (sterile latex gloves, medical/surgical masks, gowns/aprons, and eye protection) than private facilities (70.6% vs. 66.5%; 64.7% vs. 49.2%; 61.8% vs. 44%; and 11.8% vs. 5.5%, respectively). Safe disposal of sharps and medical waste was observed in 58.8% and 73.5% of public and 61.5% and 66.7% of private facilities, respectively. Appropriate storage of sharps and medical waste was observed in a higher proportion of public facilities than private facilities (61.8% vs. 52.2%, and 67.6% vs. 66.7%, respectively). Less than half of the facilities in Herat province reported having running water, handwashing soap, environmental disinfectant, sterile latex gloves, medical/surgical masks, and eye protection equipment in the general outpatient area (Supplementary Table 1).

### COVID-19 infection in healthcare workers

Table 3 summarizes the reported COVID-19 cases in seven provinces (Balkh, Herat, Kabul, Kandahar, Kunduz, Nangarhar, and Paktya). As of November 03, 2020, Kabul province had the highest number of infected HCWs (n = 1010), followed by Herat province (n = 1008). However, the total number of infected HCWs per 100,000 population was higher in Herat province compared to other provinces.

**Table 3:** Reported COVID-19 cases among healthcare workers and non-healthcare workers by province

**Province**	**Total Population** **(2020–21)**	**Total Cases**	**Total Infected HCWs**	**Total cases excluding infected HCWs**	**Total cases/** **100K population**	**Total cases excluding infected HCWs/** **100K population**	**% Cases/** **population**	**Total Infected HCWs/** **100K population**	**% Infected HCWs/** **total cases**
Balkh	1,509,183	2402	245	2157	159	143	0.16%	16	10.20%
Herat	2,140,662	7114	1008	6106	332	285	0.33%	47	14.17%
Kabul	5,204,667	15,306	1010	14,296	294	275	0.29%	19	6.60%
Kandahar	1,399,594	1648	82	1566	118	112	0.12%	6	4.98%
Kunduz	1,136,677	734	35	699	65	61	0.06%	3	4.77%
Nangarhar	1,701,698	1471	348	1123	86	66	0.09%	21	23.66%
Paktya	611,952	1244	51	1193	203	195	0.20%	8	4.10%

**Total**	**13,704,433**	**29,919**	**2779**	**27,140**	**1257**	**1137**		**120**	

**Note:** Data were obtained from Ministry of Public Health data warehouse updated as of November 03, 2020. 100K = 100,000; HCWs, Healthcare workers.

Overall, HCWs had an infection rate of 120 per 100,000 population compared to non-HCWs of 1137 per 100,000 population (9 times lower) in the targeted seven provinces.

### Total COVID-19 infected HCWs per 100,000 population by WASH indicators and standard precautions in HCFs

**Fig. 2:**
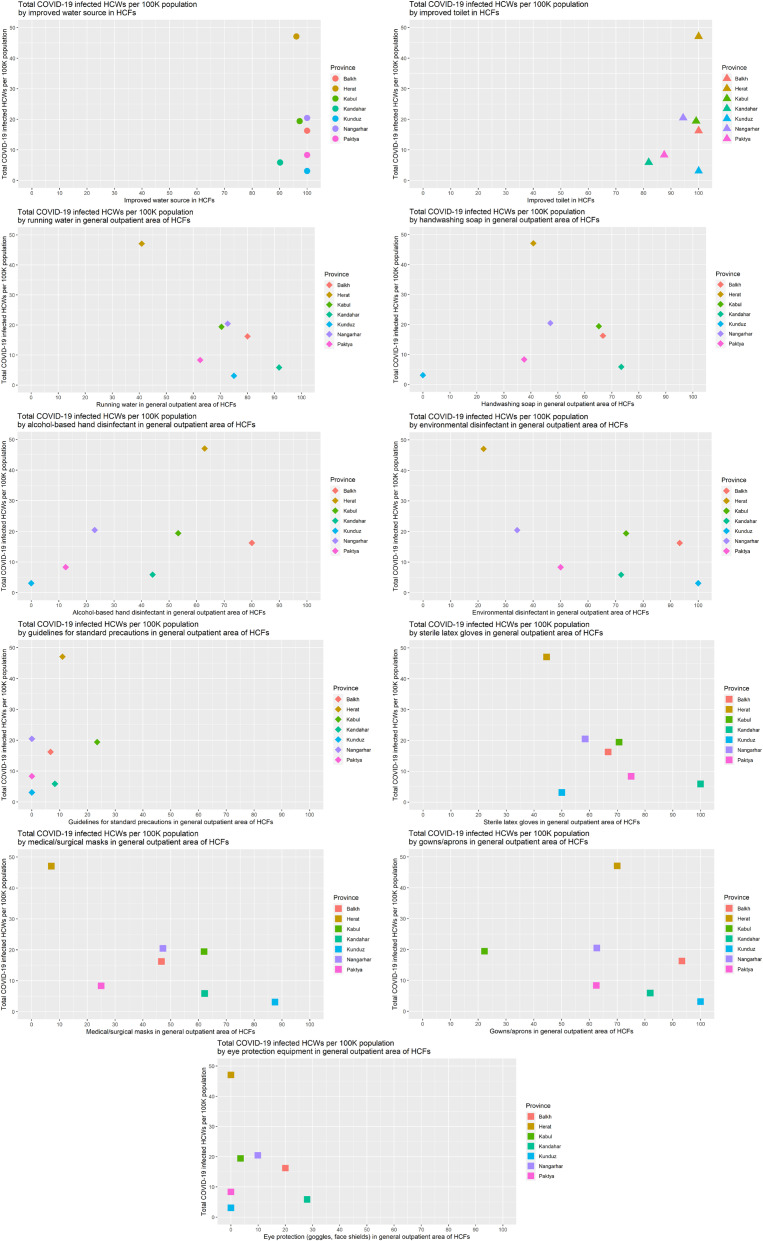
Plots of total COVID-19 infected HCWs per 100K population against WASH indicators and standard precautions in HCFs

Of the seven provinces, Herat had the highest cumulative COVID-19 case rate among HCWs per 100,000 population and reported a lower availability of running water, environmental disinfectant, sterile latex gloves, medical/surgical masks, and eye protection equipment (goggles, face shields) in the general outpatient area of HCFs compared to other provinces. In contrast, with the lowest cumulative COVID-19 case rate among HCWs per 100,000 population, Kunduz province reported a higher availability of improved water sources, improved toilets, environmental disinfectant, medical/surgical masks, and gowns/aprons compared to other provinces. Kandahar province, with the second lowest cumulative COVID-19 case rate among HCWs per 100,000 population, reported a higher availability of running water, handwashing soap, sterile latex gloves, medical/surgical masks, and eye protection equipment compared to other provinces (Fig. 2).

### Geospatial distribution of reported COVID-19 cases and infected HCWs by total population and province

**Fig. 3:**
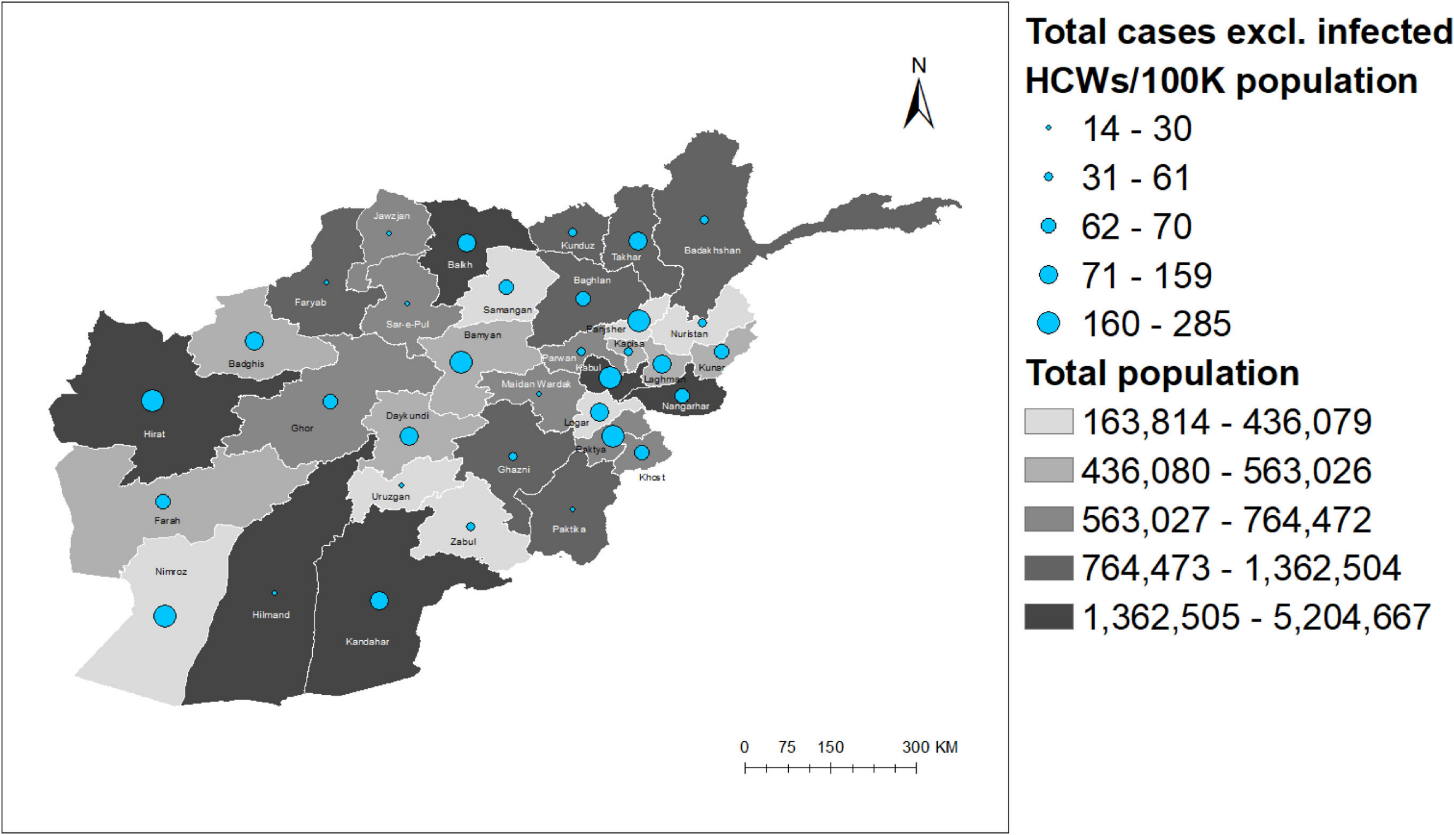
A map of Afghanistan showing the distribution of total COVID-19 cases excluding infected HCWs per 100,000 population by provinces from February 24, 2020 to November 03, 2020.

**Fig. 4:**
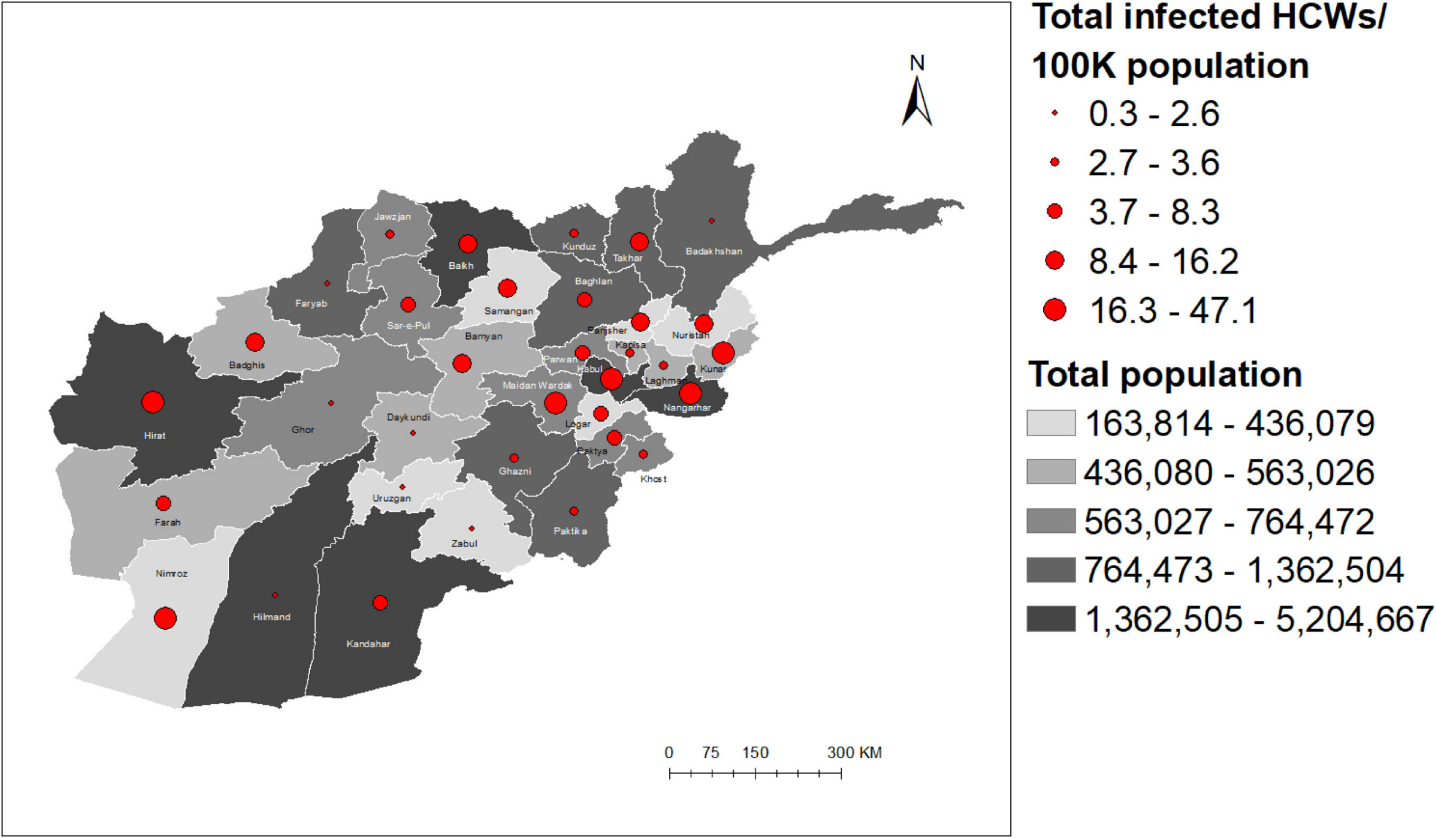
A map of Afghanistan showing the distribution of total infected healthcare workers per 100,000 population by provinces from February 24, 2020 to November 03, 2020.

Geospatial distribution demonstrated a different pattern of total COVID-19 cases (excluding infected HCWs) and infected HCWs per 100,000 population per province. Of the seven provinces included in the study, two provinces with larger populations (Herat and Kabul) had higher COVID-19 cases per 100,000 population; however, Nangarhar, Kandahar, and Kunduz provinces had relatively lower COVID-19 cases per 100,000 population, despite a large population. On the other hand, Paktya province, with the lowest population among the provinces selected, had relatively higher COVID-19 cases per 100,000 population (Fig. 3). In terms of infected HCWs per 100,000 population, Herat province, with the second largest population in the list, had the highest number of infected HCWs per 100,000 population, followed by Nangarhar and Kabul provinces (Fig. 4). Kunduz province reported the lowest number of infected HCWs per 100,000 population in the list of seven provinces. In Figs. 3 and 4, the population of provinces is shown in white-to-grey gradient while that of total COVID-19 cases and total infected HCWs per 100,000 population is shown as blue and red circles, respectively. The darker the color, the larger the population, and the larger the circle, the greater the total number of COVID-19 cases and infected HCWs per 100,000 population.

**Fig. 5:**
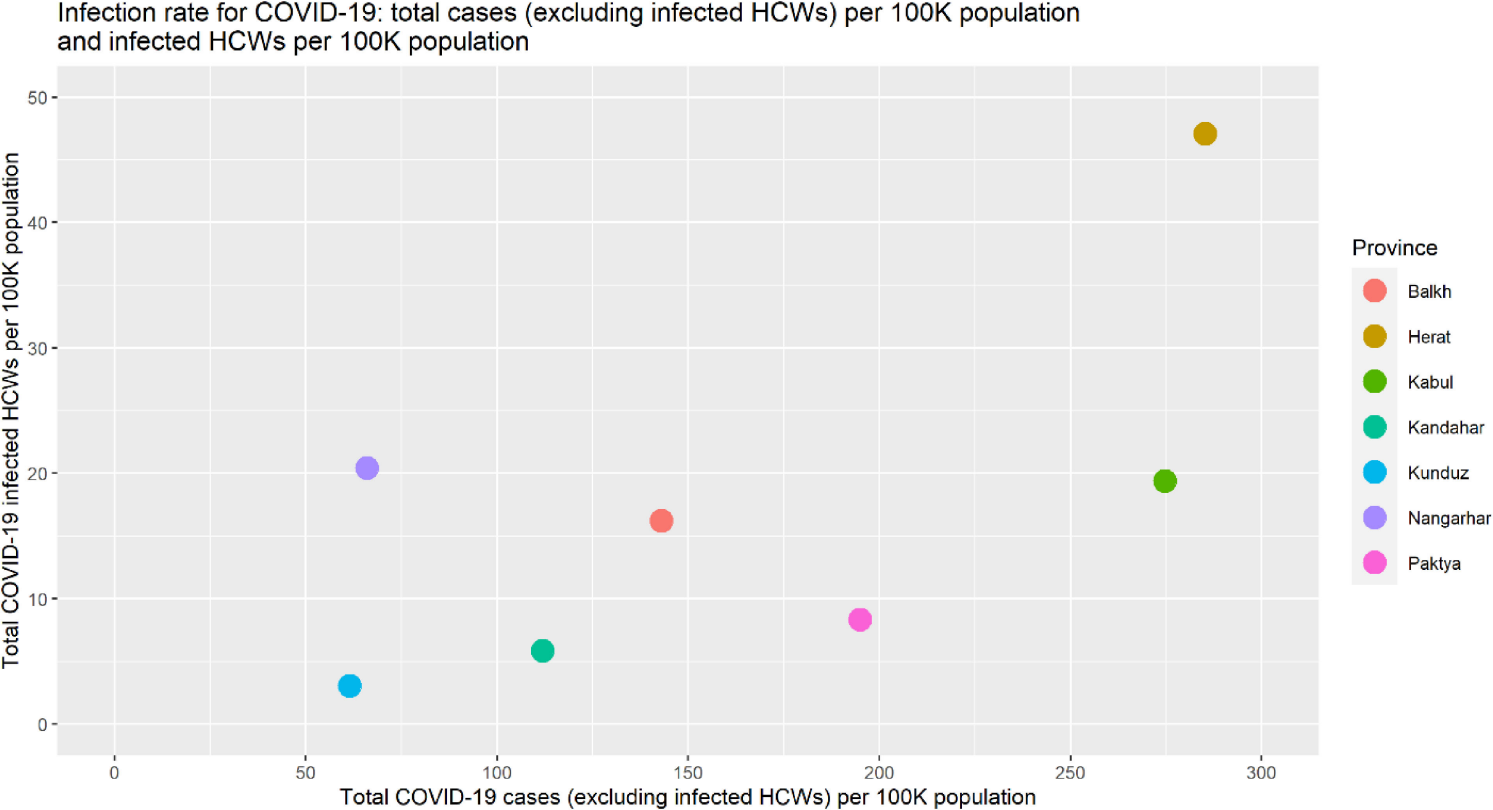
Scatter plot of total COVID-19 cases excluding infected HCWs and total COVID-19 infected HCWs per 100,000 population per province from February 24, 2020 to November 03, 2020.

Figure 5 illustrates a moderately strong positive correlation between the total COVID-19 cases (excluding infected HCWs) per 100,000 population and the total infected HCWs per 100,000 population. In comparison to the total COVID-19 cases per 100,000 population, the total infected HCWs per 100,000 population was relatively higher in Nangarhar province and lower in Paktya province than in other provinces.

## Discussion

The survey data revealed that more than 97% of Afghanistan’s HCFs used an improved water source and improved toilets. This figure is higher than the global estimates in 2019 that 74% of HCFs used an improved water source [6]. An assessment of HCFs in LMICs revealed that 50% of HCFs lack on-site piped water and 33% had no improved toilets [2]. A systemic review designed to assess the water availability at hospitals in LMICs reported that only 66% of hospitals had water availability [16]. Insufficient availability of WASH services limits the delivery of routine healthcare, including performing safe childbirth, performing safe surgical procedures, cleaning, and hand hygiene in HCFs [7, 17–19] and poses health risks to the visitors and medical and non-medical staff of HCFs [17]. In addition, the provision of improved water sources and proper sanitation and hygiene services might be critical in combating COVID-19. Securing the basic water and sanitation services in HCFs is critical to delivering safe and quality healthcare and reducing the risk of infection. National policies should prioritize the availability of water and sanitation services in HCFs and allocate funds for the construction and maintenance of these essential infrastructure resources at all levels of HCFs.

Our study also found a low availability of hygiene services and standard precautions in HCFs that must be addressed. A previous assessment found that only 2% of HCFs in LMICs with data had adequate WASH and waste management services and standard precautions [2]. In our study, of the seven provinces surveyed, Herat province had the highest cumulative COVID-19 case rate among HCWs per 100,000 population and reported inadequate availability of WASH services and standard precautions in HCFs. Given the constraints of the limited resources to implement key IPC measures in LMICs, HCWs have a high risk of contracting COVID-19. According to World Health Organization, over 10,000 HCWs in 40 African countries have been infected with COVID-19 as of July 2020, where many HCFs were found to lack the core infrastructure required to adopt key IPC measures [20]. This may indicate that inadequate availability of the IPC measures in HCFs contributes to an increased risk of infection among HCWs, and precise information on these would help to make better decisions and better protect health [2]. Due to inadequate hygiene services and standard precautions, infection prevention measures are not fully implemented in HCFs within LMICs. Pre-pandemic preparedness is central to an effective response and recovery strategy and has potential implications for addressing priorities for future public health emergencies. A comprehensive review on WASH and COVID-19 transmission suggested incorporating WASH services into response and recovery plans and integrating the WASH sector with other relevant sectors to enhance prevention efforts [12]. Most WASH efforts are centered on reducing the spread of infection during a pandemic; however, the pre-pandemic stage also provides opportunities for WASH efforts to decrease the risk of infection during the pandemic stage [12]. Furthermore, investing in WASH services in HCFs is one of the most cost-effective strategies to improve pandemic preparedness, particularly in low-resource settings [3]. Administrators at HCFs are recommended to use these insights to prepare comprehensive preparedness and response plans that include information on the availability of WASH services and standard precautions to minimize the healthcare-associated transmission of infections, especially during outbreaks.

The MoPH in Afghanistan designed the Health Center Hygiene Program (HCHP) in collaboration with United Nations Children’s Fund (UNICEF) in 2017 and it has been implemented in close coordination with non-governmental organizations. The HCHP is based on hygiene behavior change within health centers by providing WASH services and triggering healthcare providers to adopt hygiene and sanitary practices. In 2020, the program was implemented in 200 health centers in five provinces (Herat, Kandahar, Badakhshan, Nangarhar, and Bamyan), and many of these health centers were declared as Model Health Centers (MHC) for WASH. The health centers are evaluated based on the proposed criteria set for MHC, including the availability of WASH services and regular hygiene and sanitary behaviors by health center personnel. Scaling up the program for wider implementation may help address the existing problems regarding the lack of WASH services in health centers that put healthcare providers and clients at higher risk of infections.

Our findings reveal the extent to which WASH services and standard precautions were available in HCFs before the COVID-19 pandemic and provide evidence on the relevance of WASH services and standard precautions with COVID-19 infected HCWs. The COVID-19 pandemic and other disruptions have significantly altered the delivery of healthcare worldwide. Enhancement in infection prevention and control measures is considered essential to ensuring that health systems can remain fully functional with rapid responsiveness. Therefore, HCF administrators are recommended to look beyond the current exigencies of COVID-19 and address the fractures in the delivery of healthcare that have been exposed by the pandemic.

Our study’s main strength is that it is the first analysis to assess the availability and readiness of HCFs for the WASH services and standard precautions in a low-resource setting by using the AfSPA 2018-19 dataset, which is a representative sample of HCFs. The AfSPA 2018-19 involved complex sampling techniques; therefore, the findings were weighted to compensate for non-response and disproportionate selection of the sample. The limitations of this study need to be noted. Data on WASH services and COVID-19 cases originated from two different sources that were collected at different timepoints. It was not applicable to investigate statistical relationships among variables as the records from both datasets could not be linked. The level or size of the HCFs was not considered in the survey; nonetheless, the availability and readiness of certain WASH services and standard precautions would vary depending on the level or size of the HCFs. This can be explored analytically in future studies. Additionally, the data were from seven major and comparatively developed provinces, and most HCFs were private hospitals and clinics. Thus, the findings should be generalized with caution to the overall HCFs.

## Conclusion

Our findings reveal disparities in the availability and readiness of WASH services and standard precautions faced by public and private facilities in Afghanistan before the COVID-19 pandemic. Private facilities had a lower availability of hygiene services and standard precautions than public facilities. Of the seven provinces surveyed, Kunduz and Kandahar provinces with higher availability of WASH services and standard precautions in HCFs had lower cumulative COVID-19 case rates among HCWs per 100,000 population. It highlights the potential role of integrating adequate WASH services and standard precautions in emergency preparedness and response plan to prevent pandemics effectively.

## Data Availability

The datasets used during the current study are available from the DHS program website (https://dhsprogram.com/data/available-datasets.cfm) and MoPH Data Warehouse website (http://covid.moph-dw.org/#/).

## Supplementary information

**Additional file 1:** **Supplementary Table 1:** Availability of standard precautions for infection prevention and control in general outpatient areas, and healthcare waste management by province, N=142.
