# Supplementary material for: Status of water, sanitation, and hygiene and standard precautions in healthcare facilities and its relevance to COVID-19 in Afghanistan
Source: Environ Health Prev Med. 2022 Feb 23;27:6. doi: 10.1265/ehpm.21-00272 (PMC9093622; doi:10.1265/ehpm.21-00272)
Supplement: Supplementary file 1 — Additional file 1: Supplementary Table 1: Availability of standard precautions for infection prevention and control in general outpatient areas, and healthcare waste management by province, N=142. [file ehpm-27-006-s001.docx]

| **Supplementary Table 1. Availability of standard precautions for infection prevention and control in general outpatient areas, and healthcare waste management by province, N=142** | | | | | | | | |
| --- | --- | --- | --- | --- | --- | --- | --- | --- |
|  | ***Weighted n* (%)** | | | | | | | |
| **Variables** | **Balkh** | **Herat** | **Kabul** | **Kandahar** | **Kunduz** | **Nangarhar** | **Paktya** | **Total** |
| **Hygienic conditions, observed** |  |  |  |  |  |  |  |  |
| Running water | 8 (80.0) | 8 (40.9) | 54 (70.4) | 10 (91.7) | 4 (75.0) | 11 (72.7) | 3 (62.5) | **98 (68.9)** |
| Handwashing soap | 7 (66.7) | 8 (40.9) | 50 (65.2) | 8 (73.5) | 0 (0.0) | 7 (47.2) | 2 (37.5) | **82 (57.3)** |
| Alcohol-based hand disinfectant | 8 (80.0) | 12 (62.9) | 41 (53.3) | 5 (44.0) | 0 (0.0) | 3 (23.0) | 1 (12.5) | **70 (49.0)** |
| Disinfectant (environmental, e.g., chlorine) | 9 (93.3) | 4 (22.0) | 57 (73.8) | 8 (72.0) | 5 (100.0) | 5 (34.2) | 3 (50.0) | **91 (64.0)** |
| Guidelines for standard precautions | 1 (6.7) | 2 (11.0) | 18 (23.5) | 1 (8.3) | 0 (0.0) | 0 (0.0) | 0 (0.0) | **22 (15.2)** |
| **Personal protective equipment, observed** |  |  |  |  |  |  |  |  |
| Sterile latex gloves | 7 (66.7) | 8 (44.5) | 54 (70.6) | 11 (100.0) | 3 (50.0) | 9 (58.4) | 4 (75.0) | **96 (67.2)** |
| Medical/surgical masks | 5 (46.7) | 1 (7.1) | 47 (62.0) | 7 (62.2) | 5 (87.5) | 7 (47.2) | 1 (25.0) | **73 (51.7)** |
| Gowns/aprons | 9 (93.3) | 13 (70.0) | 17 (22.3) | 9 (81.9) | 5 (100.0) | 10 (62.7) | 3 (62.5) | **66 (46.8)** |
| Eye protection (goggles, face shields) | 2 (20.0) | 0 (0.0) | 3 (3.5) | 3 (28.0) | 0 (0.0) | 1 (9.9) | 0 (0.0) | **9 (6.5)** |
| **Healthcare waste management, observed** |  |  |  |  |  |  |  |  |
| Safe final disposal of sharps waste | 6 (60.0) | 17 (92.9) | 45 (58.3) | 7 (62.2) | 2 (37.5) | 7 (48.4) | 3 (50.0) | **87 (61.1)** |
| Safe final disposal of medical waste | 5 (46.7) | 17 (92.9) | 58 (75.4) | 8 (72.0) | 0 (0.0) | 6 (39.7) | 3 (50.0) | **97 (67.8)** |
| Appropriate storage of sharps waste | 5 (46.7) | 2 (11.0) | 49 (64.6) | 9 (81.9) | 3 (50.0) | 7 (44.1) | 2 (37.5) | **77 (53.8)** |
| Appropriate storage of medical waste | 9 (93.3) | 13 (62.8) | 48 (62.8) | 6 (57.4) | 5 (87.5) | 9 (62.1) | 4 (75.0) | **94 (66.8)** |
|  |  |  |  |  |  |  |  |  |

**Note.** Data were obtained from the AfSPA survey 2018-19.
